# Man versus machine: cost and carbon emission savings of 4G-connected Artificial Intelligence technology for classifying species in camera trap images

**DOI:** 10.1038/s41598-024-65179-x

**Published:** 2024-06-24

**Authors:** James Smith, Ashleigh Wycherley, Josh Mulvaney, Nathan Lennane, Emily Reynolds, Cheryl-Ann Monks, Tom Evans, Trish Mooney, Bronwyn Fancourt

**Affiliations:** 1 Kangaroo Island Landscape Board, Kingscote, SA 5223 Australia; 2 School of Agriculture and Environmental Science, University of Western Australia, Perth, WA 6009 Australia; 3 School of Environmental and Rural Science, University of New England, Armidale, NSW 2351 Australia; 4 Bush Heritage Australia, Melbourne, VIC 3008 Australia; 5 Department of Environment and Water, Government of South Australia, Kingscote, Australia; 6 Department of Environment, Science and Innovation, Queensland Parks and Wildlife Service & Partnerships, Toowoomba, Australia; 7 School of Biology and Environmental Science, Queensland University of Technology, Brisbane, Australia

**Keywords:** Ecology, Biodiversity

## Abstract

Timely and accurate detection and identification of species are crucial for monitoring wildlife for conservation and management. Technological advances, including connectivity of camera traps to mobile phone networks and artificial intelligence (AI) algorithms for automated species identification, can potentially improve the timeliness and accuracy of species detection and identification. Adoption of this new technology, however, is often seen as cost-prohibitive as it has been difficult to calculate the cost savings or qualitative benefits over the life of the program. We developed a decision tool to quantify potential cost savings associated with incorporating the use of mobile phone network connectivity and AI technologies into monitoring programs. Using a feral cat eradication program as a case study, we used our decision tool to quantify technology-related savings in costs and carbon emissions, and compared the accuracy of AI species identification to that of experienced human observers. Over the life of the program, AI technology yielded cost savings of $0.27 M and when coupled with mobile phone network connectivity, AI saved $2.15 M and 115,838 kg in carbon emissions, with AI algorithms outperforming human observers in both speed and accuracy. Our case study demonstrates how advanced technologies can improve accuracy and cost-effectiveness and improve monitoring program efficiencies.

## Introduction

Timely and cost-effective methods are necessary for monitoring species of conservation significance, invasive species control programs and protected area management^1^. Sparsely distributed or wide-ranging species are challenging to detect and monitor^2^, typically leading to significantly higher monitoring and program costs^3,4^. Over recent decades, camera traps have become one of the most widely used methods for ecological monitoring^5,6^ as they can provide a cost-effective way of continuously surveying cryptic, low density and wide-ranging species over large geographical areas for extended periods of time. Extensive camera trap networks, however, consume vast amounts of staff time to travel to and from field sites, change batteries, download data and sort through empty and unwanted images, then identify and classify target species in each image^7,8^. The ongoing time and cost of servicing camera traps and manually reviewing and classifying images typically limits the number of cameras that can be deployed on a program, and how often those camera images can be retrieved and downloaded, potentially reducing the comprehensiveness and usefulness of any monitoring data collected.

For some monitoring programs, the time lag between when an animal is detected on a camera, and the image being classified by program staff (often weeks or months later), can lead to significant costs and delays in program delivery. For example, in the context of a feral animal eradication program, the faster a feral animal detection can be communicated to on-ground eradication staff, the faster they can respond, increasing the probability that the animal can be swiftly removed, reducing ongoing labour costs and expediting program delivery. Timely and comprehensive monitoring is particularly important for eradication programs because every individual of the target species needs to be detected and removed before eradication can be declared with confidence ^9,10^.

Advanced technologies, such as machine learning for image classification and species identification, have significantly reduced the labour-intensive aspects of image processing^11–13^. New web-based platforms allow users to upload camera trap data from SD cards to the cloud, where machine learning algorithms can identify and label target species using artificial intelligence (AI)^7,14^. The use of AI image classification algorithms has additional advantages because every image is scrutinised with the same level of precision, whereas human observers can suffer from fatigue, boredom, distraction or lack of concentration when reviewing large numbers of images. However, the need to retrieve SD cards from cameras in the field before uploading images to the cloud still results in substantial lag times between an animal triggering a camera, and the user being aware of its detection.

Camera traps with 4G capability can connect to 4G mobile phone networks, enabling images to be uploaded to the cloud almost instantly. 4G-connected camera traps negate the need for staff to travel (sometimes long distances) to every individual camera location to retrieve SD cards then manually upload them to the cloud, resulting in significant savings in both staff time and travel costs. Additionally, by directly linking these 4G-connected camera traps to AI image classification algorithms, images can be instantly uploaded and species identified automatically, alerting staff to the presence of a target animal in real-time.

The higher initial and ongoing costs of advanced technologies can *prima facie* appear to be prohibitively expensive, potentially influencing program managers to continue using ‘cheaper’ traditional camera trap networks. But this could be a false economy, as the ongoing savings of travel and staff time to retrieve and download SD cards and classify images are often not taken into account, with investment decisions based solely on up-front purchase costs. Such investment decisions might also fail to consider any program delivery impacts of time lags between detection and awareness of an animal’s presence, such as delays in the control or eradication of a pest animal. To understand and evaluate the complete cost of a monitoring program, all initial and ongoing costs and savings, including travel and staff time, should be considered. Only then can any true net benefits from using advanced technologies be robustly compared with those of traditional camera trap networks for any given program.

We developed a cost–benefit decision tool to assist program managers to examine and evaluate the total cost of using different camera trap network scenarios to monitor target species across the life of their program. We included separate options for using (1) manual SD card image downloads and manual image classification, (2) manual SD card image downloads plus AI algorithms for image classification, and (3) mobile phone networks for uploading images directly from the camera to the cloud plus AI algorithms for image classification. Using a feral cat (*Felis catus*) eradication program as a case study, we used the decision tool to quantify and compare technology-related costs and savings, to those using traditional camera networks with manual image download and processing. We also compared the accuracy of the AI algorithm for species identification with that of experienced human observers.

## Methods

### Artificial intelligence algorithm and 4G network connectivity

In this study we used the eVorta online AI image classification platform (www.evorta.com) which automatically identifies and classifies target species in camera trap images with a given level of confidence. eVorta is an Australian machine learning system that utilises a three-stage convolutional neural net box detector built specifically for fixed focus camera trap images (Hamesh Shah, eVorta, pers comm). The eVorta online interface allows for customisation of classified image results (including confidence levels of each image classified), customisable time frames for interrogation, a mapping function, real time alerts (via SMS or email) for species of interest, and the ability for users to interrogate and train incorrectly classified images for continual improvement of the AI algorithms.

Cameras equipped with 4G capability can be fitted with a 4G mobile phone SIM card and configured to transmit images directly to a nominated online destination (in our case to eVorta) using the 4G network. In areas with no 4G mobile reception or where non-4G capable cameras are used, images on SD cards can be manually uploaded (or posted) to eVorta for processing.

### Decision tool

We developed a cost–benefit decision tool to provide a three-way comparison of (1) manual SD card image downloads and manual image classification, (2) manual SD card image downloads plus AI algorithms for image classification, and (3) using 4G-connected cameras to directly upload images from the camera to the cloud plus AI algorithms for image classification. The tool allows users to input program-specific costs and parameters (e.g. number of cameras, fuel usage, distances driven, program duration, etc.) to determine the most cost and/or time effective scenario for their program (Supplementary Material 3).

### Case study: Kangaroo Island feral cat eradication program

**Figure 1:**
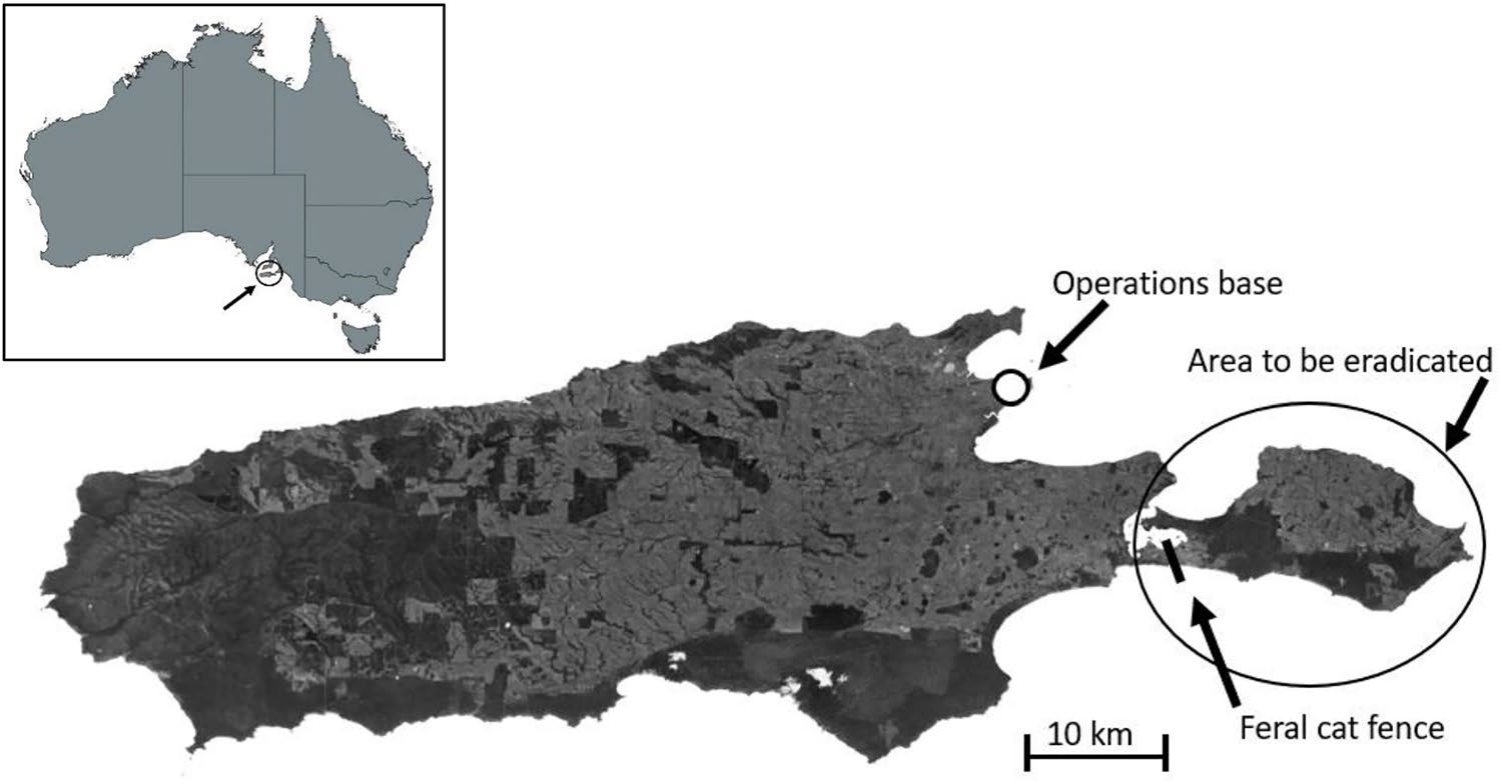
Map of Kangaroo island showing location of operations base and the area used (Dudley Peninsula) in this study. Insert shows location of Kangaroo Island in Australia. The distance (by road) from the operations base to the easternmost tip of the study area is 75 km. Map was generated using QGIS (version: 3.30.0-‘s-Hertogenbosch, https://qgis.org/en/site/forusers/download.html).

The Kangaroo Island Landscape Board’s Feral Cat Eradication Program (KIFCEP) is currently eradicating feral cats from the Dudley Peninsula on the eastern end of Kangaroo Island, Australia (− 35.8069, 137.9826). The Dudley Peninsula comprises 384 km^2^ of mixed tenure farmland and woodland, with many areas difficult to access (Fig. 1). A predator-proof feral cat barrier fence spans the width of the narrow 3.5 km wide isthmus that joins the peninsula to the rest of the island (Fig. 1). The KIFCEP is moving a rolling eradication front from east to west towards the barrier fence, creating an expanding cat-free area where any new cat incursions need to be swiftly detected and removed to prevent the population from re-establishing behind the eradication front. As the cat-free area increases, the effort required to monitor behind the front also increases.

A total of 200 fixed monitoring cameras are required to ensure adequate spatial coverage of the 384 km^2^ eradication area. Cameras are placed in likely feral cat egress locations and spaced a minimum of 1 km from the nearest camera. Individual camera sites are located between 30 and 75 km away from the operations base in Kingscote (Fig. 1). To service all 200 cameras (maintain and swap batteries and SD cards) would take up to four staff day equivalents.

1. Cost and carbon emission savings: comparison of AI image classification, 4G connectivity and traditional camera networks.
  We used the decision tool in Supplementary Material 2 to quantify costs and potential savings for the KIFCEP monitoring program, using three different scenarios.
  i) Traditional camera traps with manual image downloads and manual image classification (images recorded on SD cards, staff travel to field to retrieve SD cards from cameras, staff manually download and classify images);
  ii) Traditional camera traps with manual image downloads plus eVorta AI algorithm for image classification (images recorded on SD cards, staff travel to field to retrieve SD cards from cameras, images manually uploaded by staff to eVorta platform for automatic image classification); and
  iii) 4G-connected camera traps for uploading images directly from cameras to the cloud plus AI algoirthms for image classification (images instantly sent via the 4G mobile network from the camera to the eVorta platform for AI image classification).
  The traditional camera traps comprised a Swift Enduro camera (non-4G-capable; Outdoor Cameras Australia, Toowoomba, QLD, Australia) with an on-board SD card to record images. The camera was powered using 12 AA batteries. While optional external solar panels (with their own battery) can be fitted to these cameras to provide more battery life (not charge the AA batteries), we did not include this option in our analysis. As SD cards still need to be regularly retrieved from these cameras, the use of a solar panel would not provide any time or cost savings, as batteries can be changed at the same time as the SD card is retrieved. Accordingly, the solar panel would represent an additional cost with no time or cost savings.
  The 4G-capable camera traps comprised a 4G-capable Swift Enduro camera (Outdoor Cameras Australia, Toowoomba QLD, Australia) with an on-board 4G mobile phone SIM card. Cameras located in areas with weak 4G signal were fitted with long-range antennas to improve connectivity. The cameras were powered using 12 AA batteries that were supplemented by an externally fitted solar panel with inbuilt battery, negating the need to regularly service cameras to change batteries. In our analyses we assumed batteries and SD cards where changed each time well before they were anticipated to be flat or full, respectively, based on observations during the field trial.
  Comparisons of cost and time were made assuming continuous running of camera traps for three years. Input values were either derived from actual costs and time recorded by the KIFCEP, or from manufacturer’s published mean values (e.g., for vehicle fuel consumption). Initial hardware purchases were included as a one-off cost. The costs associated with the initial deployment of cameras (driving to sites) were assumed to be the same for each scenario, and hence were not included in our comparative analysis.
  We used an image download frequency of ‘daily’ because in the KIFCEP, timely notification of target species (feral cats) walking past cameras was paramount.
  The carbon emissions for the make and model of vehicle used on the program were taken from the vehicle manufacturer’s website.
2. Species identification classification: human versus machine
  To compare the accuracy of the eVorta AI algorithm system of species classification against trained staff, we uploaded 101,586 images to eVorta that had been sorted and classified by field staff using ExifPro 2.1.0.2^15^. Images were collected from a subset of 42 camera traps between 11 November 2020 and 9 September 2021. None of the images in this set had previously been sent to eVorta, so they represented a novel set of images for comparison to the AI algorithm.
  For images of feral cats and the Kangaroo Island echidna (*Tachyglossus aculeatus multiaculeatus*), we compared the human-tagged image results with those classified by eVorta. We quantified several comparisons to examine the completeness and accuracy of eVorta as an image recognition tool, specifically:
  i) the number of images correctly classified by eVorta but missed by humans,
  ii) the number of images correctly classified by humans but missed by eVorta,
  iii) the number of image trigger sets correctly classified by eVorta but missed by humans, and
  iv) the number of image trigger sets correctly classified by humans but missed by eVorta.
  An image trigger set was defined as the three images captured for each camera trigger. Sequences were defined as a group of consecutive trigger sets where an animal remains in front of the camera. Consecutive trigger sets of the same species that were separated by at least 5 s were classified as separate sequences. As long as the recorder (human or machine) correctly classified at least one image in the sequence, the entire sequence was recorded as correctly classified. Using these metrics, we then compared the type I (false positive) and type II (false negative) error rates between eVorta- and human-classified images and sequences.

## Results

All dollar amounts presented herein are in Australian dollars ($AUD).

### Decision tool

Our cost–benefit decision tool is provided in Supplementary Material 3. The tool allows for multiple inputs which can be manipulated to determine the most cost-effective monitoring scenario (whether to use AI, with or without 4G connectivity) for any given program.

### Case study: Kangaroo Island feral cat eradication program

1. Cost and carbon emission savings: comparison of AI image classification, 4G connectivity and traditional camera networks.
  Using traditional camera traps with manual image downloads and processing, monitoring costs for the KIFCEP totalled $2.67 m over the 3-year program (Table 1). Staff costs accounted for the majority of this ($2.10 m, 79%), reflecting the significant amount of staff time needed to drive to camera locations, retrieve SD cards and manually classify images (Supplementary Material 2). The use of eVorta to classify images saved $0.27 m (10%), while the use of 4G-connected cameras combined with eVorta yielded cost savings of $2.15 m (81%) over traditional camera traps requiring manual image download and processing (Table 1). While the cost of processing images by eVorta was an additional expense, the savings in staff time and travel costs more than offset the additional processing cost.

**Table 1:** Annual savings using a 4G-connected Artificial Intelligence based image recognition system for classifying camera trap images when compared to the equivalent effort of field staff-based collection and tagging. Refer to Supplementary Material 2.

	Cost ($)	Saving ($)	Saving (%)
Years in the project	3		
Manual image download, human image processing	2,666,628		
Manual image download and upload, eVorta image processing	2,392,290	274,338	10.3
4G-connected camera image direct upload, eVorta image processing	519,652	2,146,976	80.5
  The 4G-connected camera network saved 115,838 kg (99%) of carbon emitted over the three-year program when compared to the non-4G-connected scenario (Supplementary Material 2).
2. Species identification classification: human versus machine
  In total, the dataset contained 492 cat images (0.5% of all 101,586 images) across 68 sequences (Table 2). One image was classified by eVorta as a cat with 94% confidence, but human observers could not identify the species with any confidence. Accordingly, this image was removed from all comparisons.

**Table 2:** Number of images, sequences and errors classified by eVorta and humans.

Species	Total number	eVorta correctly classified	Type I	Type II	Human correctly classified	Type I	Type II
Cat (images)	492	482	2751	10	469	5	18
Cat (sequences)	68	67	0	1	57	5	11
Echidna (images)	155	155	500	0	108	0	47
Echidna (sequences)	21	21	0	0	14	0	7
  eVorta correctly classified more of the cat images (n = 482, 97.9%) than humans (n = 469, 95.3%) and had fewer false negatives (n = 10) than humans (n = 18). However, humans incorrectly classified fewer images (n = 5) as a cat than eVorta (n = 2751) (Table 2). Humans missed 11 cat sequences while eVorta missed only one cat sequence. Humans also recorded five images as cats (from five sequences), which upon inspection were empty (false positives). See Table 2 for comparison. The vast majority of cat images classified by eVorta (431, 89.4%) were classified with ≥ 90% confidence. Most of the remaining 10.6% images were classified with ≥ 70% confidence (Fig. 2).

**Figure 2:**
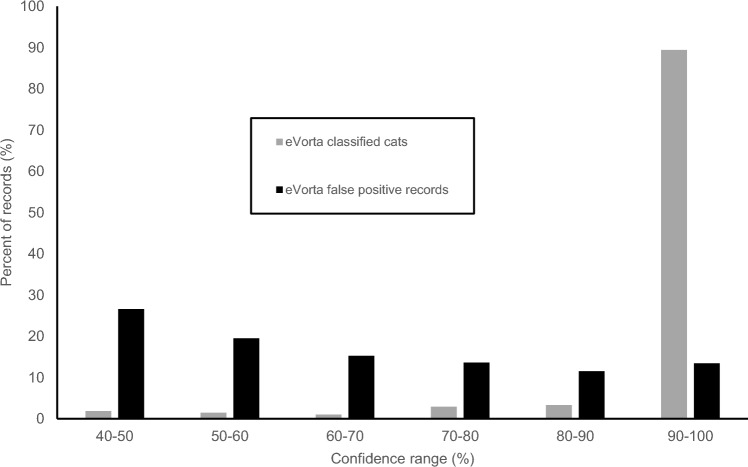
Histogram of the percent (%) of cat images eVorta classified correctly (grey) and percent of false positives (black) across all confidence levels. No images were classified as cat with ≤ 40% confidence.
  In total, the dataset contained 155 echidna images (0.2% of all images) across 21 sequences. Humans correctly classified 108 images (69.7%) but missed 47 (none were incorrectly classified). Humans also missed seven echidna sequences. Evorta missed no echidna images, however it incorrectly classified 500 false positives (Table 2). Most false positives were images of grasstrees (*Xanthorrhoea semiplana tateana*) which are very similar in form to an echidna (see Supplementary Material 2) or close-up images of the sides of sheep. All echidna images classified by eVorta were classified with ≥ 80% confidence, with all but one image classified with ≥ 90% confidence. False positives were classified with between 40 and 100% confidence (Fig. 3) with most errors occurring for images classified with above 90% confidence.

**Figure 3:**
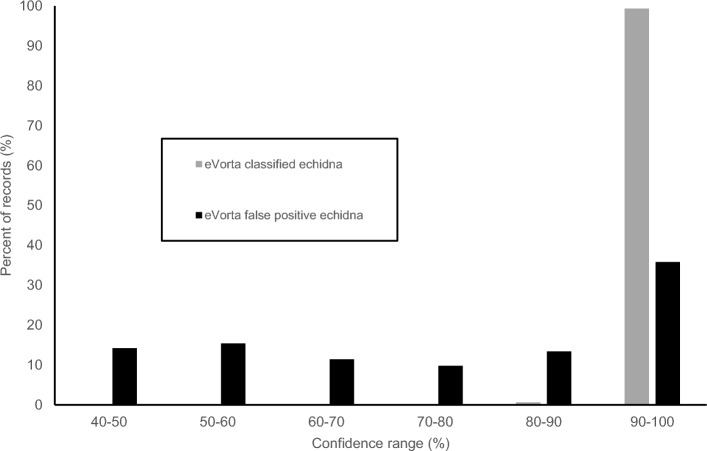
Histogram of the percent (%) of echidna images eVorta classified correctly (grey) and percent of false positives (black) across all confidence levels. No images were classified as echidna with ≤ 80% confidence.

## Discussion

Camera trap-related advanced technologies have the potential to improve the cost and timeliness of monitoring for conservation and management programs. The AI algorithm used in our study was demonstrably faster, cheaper and more accurate at image classification than humans. Coupled with 4G-connected cameras and solar panels, our monitoring system yielded significant cost savings over traditional camera trapping methods, with detections available to program staff as soon as a target species was recorded. Our cost–benefit decision tool can assist program managers to evaluate the cost-effectiveness of incorporating advanced technologies such as AI and 4G-connectivity into traditional camera trap-based monitoring programs.

### AI image classification algorithms

Our findings are consistent with several other studies, demonstrating that AI based systems are vastly more efficient and cost effective at filtering through large image arrays to identify target species, than a human based system^16,17^. While AI algorithms provide many efficiencies for identifying species in camera trap images, even the removal of empty images from datasets can represent a substantial saving^8,16^. Furthermore, because the AI system is less variable, it derives no errors from fatigue, boredom, rushing, glancing over dark images, variation between observers, or tagging images incorrectly. Almost all images that were missed by humans in our study appeared to be due to one or more of these types of errors. For example, the majority of missed cats in human-tagged images were in full view and unambiguous to the human eye, suggesting that humans likely detected but failed to tag them, or were distracted or fatigued and missed them. The eVorta system was found to have fewer errors of this type at both the individual image and at the sequence level; as well as looking at each image with ’fresh eyes’ every time, the AI internal code forces it to classify each image appropriately. The ability of AI algorithms to be trained means that image classification systems are continuously learning, leading to improvements in classification accuracy and increased species repertoires over time.

Observer bias can potentially influence the reliability of species detection and identification in camera trap images. Many methods have been used to try to quantify and minimise human-induced observer bias, such as alternating through multiple observers, or keeping observers constant^18,19^. However, these methods can be challenging for monitoring programs with tight timeframes or limited staff resources. Here, we use type I and type II errors as an objective way to quantify observer bias for both human and machine observers. Although error values will likely vary between datasets, programs and time periods, they can still be a useful reference point for evaluating the reliability or efficacy of a particular observer in any given monitoring program. For programs where the cost or time saving benefits of using AI species classification are negligible, assessment of type I and type II error rates during the early stages of the program may help to decide whether staff can still provide the required degree of accuracy when classifying camera images, or whether AI algorithms provide more accuracy for negligible cost differences. What constitutes an acceptable error rate will depend on the objective of the monitoring program. For example, for a species eradication program such as the KIFCEP used in the current study, even a low type II error rate might have significant negative delivery consequences, as every individual that goes undetected increases the cost and time taken for the complete removal of all individuals, and missed individuals could ultimately result in program failure.

An advantage of using an AI classification system that assigns confidence levels to classified images, is that not all images need to be examined by a human for verification and training. For example, from this study and for this monitoring program, examining all images classified as cats above the 40% confidence level by the eVorta system should eliminate the possibility of a feral cat being missed (as no cats were identified with < 40% confidence in this study), and would only require 3233 images classified as ‘cat’ to be reviewed. For non-target species such as the echidna, only reviewing the 655 images with a confidence level above 80% would suffice (as all echidna classifications by eVorta were made with > 80% confidence). In contrast, if looking at all images in this dataset without any AI assistance, a human would have to look through all 101,586 images in the dataset in order to classify every echidna a cat image. With further user training of the eVorta algorithm, the number of false positives will likely decrease over time, further reducing the number of images requiring manual review, and increasing the time saving when compared with human observers with no AI assistance.

While there are clear benefits and savings that can be gained from the use of AI algorithms for image processing, there are some limitations that need to be considered. Issues with model transferability between study sites or regions have been identified for some AI algorithms^20^, where algorithms trained on species in one region are not as effective when used on the same species in different regions. The eVorta algorithm used in the current study has been, and continues to be, trained on many different species from different regions across Australia (Hamesh Shah, eVorta, pers com), with new species regularly being added to the species repertoire. eVorta restricts training of the algorithm on any species to no more than 10% of total training images from any particular region, thereby increasing its exposure to varied backgrounds, vegetation and light conditions, reducing site-specificity and increasing model transferability. All of the images used for comparing human vs eVorta image classification in the current study, however, were ‘out-of-sample’ data, that is, the system had never been subjected to the background images of our dataset before, which presents a greater challenge for machine learning algorithms ^21,22^. Users of different AI image classification algorithms will need to seek a thorough understanding of the regional training and transferability of the algorithm they plan to use, and potentially factor in additional training time in their intended region or study area to overcome any transferability issues.

Another consideration when deciding to use AI algorithms is the security of the image data. Considerations include where the data travels once transmitted (sometimes via other countries), in which country they are stored, and who has access to the data during transit and/or storage^23^. The eVorta system used in the current study minimises security issues by sending all data to an Australian internet protocol address through an Australian network, however this is not the case for other commonly used AI algorithms. eVorta includes additional security measures, such as automatically blurring images of vehicles and of people to protect privacy if required. Such security and privacy aspects should be thoroughly understood before using any AI algorithm.

### 4G network connectivity

The time savings associated with using an AI image classification system can be translated directly into cost savings, but when coupled with 4G camera connectivity, cost savings can increase by orders of magnitude. Several studies have demonstrated the time ^12,13^ and cost savings associated with using machine learning for image classification. For example one previous large scale study^16^ estimated that 8.4 years (99.3%) of human related image classification work had been saved using an automatic detection system. Other studies have examined the cost savings of using AI algorithms to process images, with some estimating cost savings of up to 40%^16,17^. However, in each of these studies, the SD cards still needed to be collected from the field, requiring additional staff and travel costs, which are typically not included in their cost analysis. As we have demonstrated in the current study, the costs associated with staff travelling to camera sites to collect and download SD card images could be reduced with the use of 4G-connected cameras. Depending on the geographic area and duration over which a program operates, these cost savings could be significant. For example, while the use of AI image processing in the current study yielded a 10.3% cost saving, the use of 4G camera connectivity increased savings to over 80% (Table 1; Sup Info 2). While the cost of salaries typically increases annually, the increased use of cameras and uptake of online AI image classification systems are predicted to decrease per unit costs over time, making the technology more accessible and cost-effective than staff and vehicle costs over time.

In addition to direct cost savings, there are a range of other benefits that 4G-connected monitoring systems provide, such as the ability to respond to species detections in real time. For example, in an eradication program such as the KIFCEP, shooters or detector dogs could be immediately deployed to the camera location as soon as a feral cat is detected, increasing the chance that the cat can be swiftly removed, before it travels away from the detection area. 4G-connected systems can also facilitate the quick rectification of maintenance issues that could go unresolved for extended periods of time in non-4G-connected systems. For example, if a camera stops sending images or has shifted its field of view (or is producing too many empty images), these issues can be detected immediately through the online AI interface and remedied quickly, preventing the loss of valuable data.

There are several limitations with 4G connected camera networks that need to be considered when deciding whether or not to adopt the technology in a monitoring program. Issues can arise with 4G connected cameras when poor camera placement results in lots of ‘false trigger’ or ‘empty images’, such as those that can arise when vegetation continually triggers the camera. Trying to upload high volumes of images via File Transfer Protocol (FTP) can prematurely consume data usage plans on 4G network SIM cards, precluding further real-time image uploads over the 4G network. In many cases, proper camera placement^24^ can alleviate this. However, in situations where data plans are fully consumed, or 4G connectivity is lost, the camera’s SD card will still capture any untransmitted images. While this data cannot be reviewed in real time, it can still be recovered when the fault is detected and the SD card is manually downloaded. Whilst we did not examine this in our study, continuation of his work should include the comparison of SD card images with those transmitted, especially in areas of unreliable coverage. Cameras connected to solar panels still have facility to install AA batteries, so that power will still be available should a solar panel fail. The solar panel/external battery units used in this study were also equipped with long power cables, providing the ability to place the panels in more sunlit areas, for situations where the camera was placed in deep shade.

While 4G-connectivity can clearly improve cost efficiencies for monitoring programs, the lack of 4G network coverage in many remote areas limits its widespread adoption. For areas with sparse or sporadic coverage, range extender antennas can be added to each unit for around $130. There are also other options currently in development (Hamesh Shah pers com); with the ability to daisy-chain signals across devices, or the use of satellite connectivity to transmit images, the need for continuous 4G network coverage will soon be rendered unnecessary and make the technology available to significantly more programs than the limited 4G network currently allows.

## Conclusion

The ever-accelerating advancement and use of new technologies is providing the opportunity for larger scale and more audacious conservation-focussed programs to be carried out than ever before. In our case study, the use of AI and 4G network connectivity has enabled significant cost efficiencies and environmental benefits associated with our camera-based monitoring program, leading to a more timely and effective eradication program. Broader implications for this technology are boundless, for example real time alerts could be deployed for full time protection of threatened species nest sites, or comprehensively trained AI algorithms will be able to gather longitudinal datasets on species assemblages across vast areas with little effort from human researchers. Our decision tool will help program managers evaluate the cost effectiveness of incorporating these advanced technologies into existing or potential monitoring programs, and thus improve outcomes for conservation and management on a large scale.

### Supplementary Information

Supplementary Information 2.

Supplementary Information 3.

Supplementary Information 4.

## Data Availability

All pertinent data are found in the figures and tables. Requests for data and additional information should be submitted to the corresponding author.
