# Supplementary figures and images for: Man versus machine: cost and carbon emission savings of 4G-connected Artificial Intelligence technology for classifying species in camera trap images
Source: Sci Rep. 2024 Jun 24;14:14530. doi: 10.1038/s41598-024-65179-x (PMC11196731; doi:10.1038/s41598-024-65179-x)

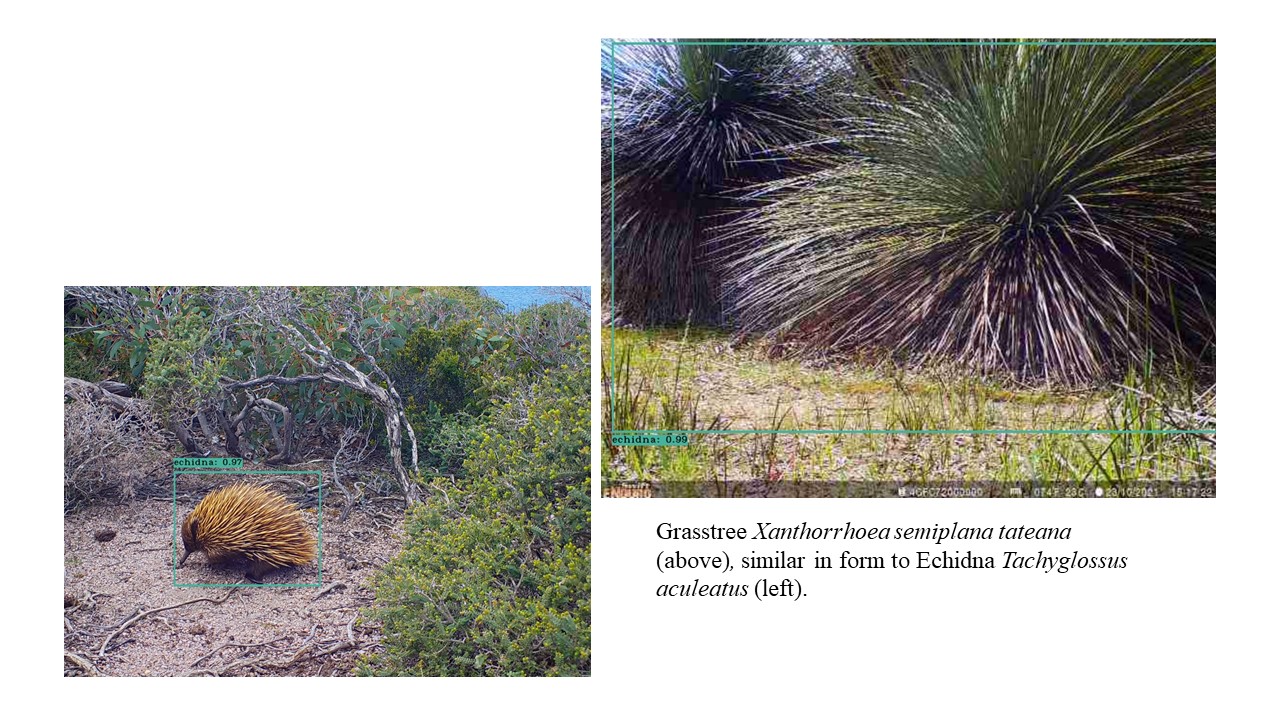

Supplement: Supplementary file 1 — Supplementary Information 2. [file 41598_2024_65179_MOESM1_ESM.jpg]
