# Supplementary material for: Man versus machine: cost and carbon emission savings of 4G-connected Artificial Intelligence technology for classifying species in camera trap images
Source: Sci Rep. 2024 Jun 24;14:14530. doi: 10.1038/s41598-024-65179-x (PMC11196731; doi:10.1038/s41598-024-65179-x)
Supplement: Supplementary file 2 — Supplementary Information 3. [file 41598_2024_65179_MOESM2_ESM.pdf]

Is This Cost Benefit Analysis per (please select) | Year

Staff Collecting and Sorting SD Card Images		Insert Values	Units
Camera Equipment Purchase Cost			
Cost per unit Camera Equipment		400	*Swift Enduro plus batteries and SD card*
Number of Cameras Required		200	
**Total Camera Equipment Purchase Cost**	**$**	**80,000**	Total initial outlay
Travel to Service Cameras - Vehicles			
Kms		140	Per vehicle per day
Fuel Price		2.45	$ per litre
Vehicle Fuel Efficiency		11.10	Litres per 100km
	**$**	**38.07**	Daily fuel cost per vehicle
Other vehicle costs:			
Vehicle Maintenance/Insurance/Registration		5.00	$ per day
Lease/Hire/Finance Expense (leased vehicles)		31.00	$ per day
Vehicle Depreciation (owned vehicles)		0.00	$ per day
	**$**	**74.07**	Total daily cost per vehicle
Number of Days Vehicle Use		365	
Number of Vehicles Used Per Day		4	*Toyota Hilux 2022*
**Total Vehicle Cost - Servicing**	**$**	**163,733**	Per Year
Travel to Service Cameras - Staff			
Staff FTE Time		7.5	Hours per day
Staff Salary Including Oncosts		52.19	$ per hour
Number of FTE Staff		4.0	Per day
**Total Staff Cost - Servicing**	**$**	**571,481**	Per Year

Image Processing - Staff			
Number of SD Card Downloads per Year		365	
Number of SD Cards to be Checked per Download		200	
Average Time Taken to Process One SD Card		2	Minutes
**Daily Staff Time - Image Processing**		**6.7**	Hours
**Total Staff Cost - Image Processing**	**$**	**126,996**	Per Year
**Total Cost of Manual Download and Processing**	**$**	**862,209**	Per Year

	Cost ($)	Saving ($)	Saving (%)
How Many Years (if Multi-year Project, or else 1)	3		
**Manual image download, human image processing**	**2,666,628**		
**Manual image download and upload, eVorta image processing**	**2,392,290**	**274,338**	**10.3**
**4G-connected camera image direct upload, eVorta image processing**	**519,652**	**2,146,976**	**80.5**

Using eVorta AI System with 4G Connected Car		Insert Values	Units
Cost per unit Camera Equipment		650	*Swift Enduro 4G plus batteries, SD card and solar panel*
Number of Cameras Required		200	
Cost of Long Range Antenna		130	$ per antenna
Number of Long Range Antennas Required		10	
**Total Camera Equipment Purchase Cost**	**$**	**131,300**	Total initial outlay
SIM Card Subscription Cost		0.60	$ per day per camera
eVorta Subscription Cost		1.00	$ per day per camera
Number of Cameras		200	as defined in cell G6
Staff Time (eVorta Liaison and Training the eVorta algorithm)		20	Minutes per day
Staff Salary Including Oncosts		52.19	$ per hour
**Total eVorta and Staff Processing Cost**	**$**	**337**	$ per day
Number of Days		365	Per Project
**Subtotal (Direct Image Uploads and eVorta Interro**	**$**	**123,150**	Per Project
Camera Maintenance Days		16	Per Project
Staff Salary Including Oncosts		52.19	Per hour
Staff FTE Time		7.5	Hours per day
Kilometres per Day		140	
**Subtotal Camera Maintenance Costs**	**$**	**6,301**	Per Project
**Total Ongoing Costs**	**$**	**129,451**	Per Project

Using eVorta AI System Without 4G Connected	Insert Values	Units
Cost per unit Camera Equipment	400	*Swift Enduro plus batteries and SD card*
Number of Cameras Required	200	
**Total Cost of Camera Purchase**	**$ 80,000**	
Number of Images	8,000	Per upload
eVorta Costs per Image	0.01	Current eVorta processing cost @ $0.01/image
Number of Uploads	365	Per Project
Staff Time (eVorta Liaison and Training the eVorta System)	20	Minutes per upload and eVorta interrogation
Staff Salary Including Oncosts	52.19	$ per hour
**Total Cost per Upload**	**$ 35,550**	

Carbon Emission Savings	Insert Values	Units
Carbon Emissions for Make and Model of Vehicle	191	g / km
**Using Staff to manually download and process images**	**39,040**	**kg / year**
**Using 4G connected cameras and eVorta image proces**	**428**	**kg / year**
**Saving**	**38,613**	**kg / year**
**Saving**	**99%**	
**Overall saving across entire project**	**115,838**	**kg**
